# The changing demographic profile of eating disorder behaviors in the community

**DOI:** 10.1186/1471-2458-14-943

**Published:** 2014-09-11

**Authors:** Deborah Mitchison, Phillipa Hay, Shameran Slewa-Younan, Jonathan Mond

**Affiliations:** School of Medicine, University of Western Sydney, Locked Bag 1797, Penrith, NSW 2751 Australia; Centre for Health Research, School of Medicine, University of Western Sydney, Sydney, Australia; School of Medicine, James Cook University, Kragujevac, Queensland Australia; Department of Psychology, Faculty of Human Sciences, Macquarie University, Sydney, Australia; Research School of Psychology, Australian National University, Canberra, Australia

**Keywords:** Epidemiology, Eating disorders, Binge, Diet, Purge, Demographic, Sex, Age, Socioeconomic

## Abstract

**Background:**

The perception that eating disorders occur predominantly in young white upper-class women has been challenged. This study examined temporal differences to the demographic correlates of eating disorder behaviors over a 10-year period.

**Methods:**

Data from cross-sectional general population surveys in 1998 (*n* = 3010) and 2008 (*n* = 3034) were collected on demographics (sex, age, income, residency), current eating disorder behaviors (binge eating, extreme dieting, purging), and health-related quality of life (SF-36).

**Results:**

Below-median annual household income was associated with increased prevalence rates from 1998 to 2008 in binge eating, extreme dieting, and purging. Male sex was associated with increased prevalence rates in extreme dieting and purging. Age over 45 years was associated with increased prevalence rates in purging. In 2008 versus 1998, binge eating was associated with greater mental health-related quality of life impairment in males but not females; and greater physical health-related quality of life impairment in regional but not metropolitan areas. Extreme dieting was also associated with greater physical health-related quality of life impairment in 2008 versus 1998 in the lower but not the higher socioeconomic sector.

**Conclusions:**

Findings suggest the ‘democratization’ of disordered eating, with greatest levels of associated impairment being within marginalized demographic sectors. Implications include the need for broader intervention programs and recruitment of demographically representative samples in eating disorder research.

**Electronic supplementary material:**

The online version of this article (doi:10.1186/1471-2458-14-943) contains supplementary material, which is available to authorized users.

## Background

In a preface to the seminal text ‘The Golden Cage’, Bruch describes anorexia nervosa as a disease that ‘*affect*(s) *young and healthy girls who have been raised in privileged, even luxurious circumstances*’ [1], highlighting the predominant view of the time that eating disorders (EDs) were the domain of the young upper-class and white female. Although it is now known within the ED field that this is not the case, this early perspective has had lasting implications for the classification and wider mental health literacy of EDs [2], as well as for the development of resources to prevent, detect, and treat EDs [3, 4]. Further, although we assume that EDs are now experienced across a range of groups, a deficit of population-based research has meant that the exact demographic profile of disordered eating remains unclear. Nonetheless, our perception of who in the population is affected by EDs has broadened. Research has shown that contrary to earlier views, males [5–8], middle-aged and older people [9, 10], people of non-white ethnicity [11–15], and people from lower socio-economic backgrounds [16–18] all experience disordered eating. An important question that remains unaddressed is whether the current prevalence of disordered eating within these groups is a stable phenomenon or has resulted from an increase in more recent times. Understanding temporal shifts in the prevalence of disordered eating in a community-based sample, as well as the specific demographic sectors within which these shifts are occurring, will enable an informed and targeted approach to the design and implementation of future prevention and treatment campaigns.

In a previous study we reported findings on the prevalence of ED behaviors from two sequential population surveys conducted in 1995 and 2005 [19]. In this study we found that the prevalence of current and regular binge eating and strict dieting had increased in both men and women. We also found that the mean age of participants who reported strict dieting and purging behaviors had increased. No comparison was made to ascertain statistical differences in the rate of increase in ED behaviors between men and women, and further work is also needed to clarify within which age groups the prevalence of ED behaviors may be changing. No studies as far as we are aware have assessed how disordered eating is changing across other demographic variables, such as socio-economic status, and urban residency.

In more recent work, we have also examined the level of impairment associated with ED behaviors. In sequential surveys conducted in 1998 and 2008 we found that participants who reported regular and current ED behaviors in 1998 and 2008 had similar levels of impairment in health-related quality of life [20]. We have also found in a cross-sectional study that impairment in health-related quality of life is similar between men and women who report ED behaviors [6, 21]. It remains unknown whether changes over time in the impairment associated with ED behaviors differ according to sex or other demographic variables.

### Aims

The aim of the current study was to examine the rate of increase of ED behaviors across the demographic features of sex, age, residency (i.e. metropolitan vs. regional), and household income. A secondary aim was to assess differences over time in health-related quality of life impairment associated with disordered eating, and whether this differed across demographic variables.

## Methods

### Design

The data for this study comes from two sequential cross sectional household surveys of the South Australian population in 1998 and 2008. The Health Omnibus Survey is conducted annually by Harrison Health Research [22], under the auspices of the South Australian Health Commission, and involves face-to-face interviews of a representative sample of the South Australian population.

### Ethics statement

All participants provided verbal informed consent and the surveys were approved by the research ethics committee of the Government of South Australia, Department of Health.

### Sample selection and interview procedure

In both 1998 and 2008, metropolitan and regional “collector” districts in South Australia were identified based on a probability proportional to size sampling procedure, according to Australian Bureau of Statistics Census data collected in 1996 and 2006. Three hundred and forty metropolitan collector districts (each including about 200 dwellings) were selected from those used in the Census. For the regional sample, towns of at least 10,000 in population as well as a balanced sample of towns of at least 1000 in population were identified from the Census. Within each collector district, a starting point was randomly selected and using a predetermined process based on a “skip” pattern of every fourth household, 10 dwellings were chosen to conduct interviews in. The person to be interviewed within each dwelling was the person who was older than 15 years and had their birthday most recently. The samples were non-replacement, and up to six visits were made to conduct an interview with the designated participant. Interviews were conducted from March until April 1998 for the 1998 survey and from February until July 2008 for the 2008 survey.

### Assessments

A range of demographic and health-related questions were included in the interviews. Questions to ascertain the presence of disordered eating were based on the Eating Disorder Examination [23], a structured interview used for ED diagnosis. Participants were asked whether they regularly (at least once per week over the past three months) engaged in (i) objective binge eating (eating an objectively large amount of food with a sense of loss of control), (ii) extreme dieting (going on a very strict diet or fasting to control weight or shape), and (iii) purging (use of laxatives, diuretics, or self-induced vomiting to control weight or shape). The specific wording of the questions, which included detailed explanations of the behaviors being assessed, can be accessed in the Additional file 1 and has also been previously published in an open access journal [19].

The Medical Outcomes Study Short Form (SF-36) was used to assess health-related quality of life, version 1 in 1998 [24] and version 2 [25] in 2008. Although there are minor variations in the wording of items, it is generally accepted that scores can be compared between the versions [26]. The SF-36 produces a physical health (PCS) and a mental health (MCS) composite scale, and transformed scores have a maximum of 100 with a normed mean of 50 and standard deviation of 10. Higher scores indicate greater quality of life. The SF-36 is validated for use in Australia [27] and there are norms for Australian and South Australian populations [28, 29]. Chronbach’s alphas for the scales are reported to range between 0.82 to 0.93 [30].

### Statistical analysis

Data were weighted according to the most recent Australian Census figures (1996 Census for the 1998 survey and 2006 Census for the 2008 survey). Demographic variables were compared between survey years using χ^2^-tests and *t*-tests. Sex (male, female) and residency (metropolitan, regional) were collected as categorical information at interview. To convert age into a categorical variable, three age groups were formed: 15–24, 25–44, and ≥ 45 years. These groups arbitrarily represent early, middle, and later adulthood and also broadly map onto eating disorder onset and persistence data e.g. [31]. The original data for annual household income was coded according to $10 000 brackets. To assist analysis, income was split around the median bracket within each survey year ($30 000 in 1998; $60 000 in 2008) resulting in one variable with two levels: < median annual household income, > median annual household income. To assess the prevalence of ED behaviors according to sex, age, residency, and annual household income, χ^2^-tests were employed. Further, in order to allow for statistical controls of other ED behaviors and demographics, multivariate logistic regressions were also conducted. To statistically compare the 1998–2008 odds ratios of ED behavior prevalence between demographic sub-groups (e.g. metropolitan vs. regional), the method described by Altman and Bland [32] to compare parameter estimates from separate analyses was used. Multivariate analyses of variance (MANOVAs) were conducted using data from participants who reported each ED behavior, with MCS and PCS scores as dependent variables, and survey year and the demographic variable of interest (sex, age, residency, and income) as independent variables. In these analyses, the MCS was included as a covariate when the dependent variable was the PCS, and vice versa. Other covariates used in the multiple logistic regression and MANOVAs (except where they were a variable of interest) included age, education, income, and body mass index, which were previously found to significantly differ between the 1998 and 2008 sample [20] as well as employment and country of birth, which differed significantly between men and women. Secular demographic changes in the population (e.g. urbanization, ageing population) were not controlled for by covariance per se, as the statistical approach contrasted the change in the proportion of participants within demographic sectors rather than the absolute increase in cases.

## Results

### Demographics

The demographics of the 1998 (*n* = 3010) and 2008 (*n* = 3034) samples are summarized in Table 1. Compared to participants in 1998, participants in 2008 were more likely to be ≥ 45 years of age, be overweight, and live in a metropolitan area, and less likely to be unemployed. Additionally, as reported previously [20], sex was nearly equally distributed in 1998 and 2008; in both years the majority of participants were either married or in a defacto relationship (61.5% in 1998, 62.7% in 2008) and had been born in Australia (75.3% both years); and in 2008 (18.2%) more participants had completed a tertiary qualification than in 1998 (11.8%).

**Table 1:** **Demographics of survey participants in 1998 and 2008**

	1998 survey ( *n,* %)	2008 survey ( *n,* %)	*χ* ^*2*^ *(df)*	*p*
Sex			0.01 (1)	0.93
Male	1464 (48.6)	1479 (48.7)		
Female	1546 (51.4)	1555 (51.3)		
Age Group			26.25 (2)	< 0.001
15 - 24 years	521 (17.3)	494 (16.3)		
25 – 44 years	1150 (38.2)	996 (32.8)		
≥ 45 years	1339 (44.5)	1544 (50.9)		
Residency			18.13 (1)	< 0.001
Metropolitan	2068 (68.7)	2235 (73.7)		
Regional	942 (31.3)	799 (26.3)		
Income*			32.28 (1)	< 0.001
< median	1127 (43.9)	1254 (52.0)		
> median	1439 (56.1)	1159 (48.0)		
BMI Group*			43.81 (3)	< 0.001
Underweight (BMI < 20.0)	91 (3.3)	74 (2.7)		
Healthy weight (20.0 < BMI < 25.00)	1330 (48.6)	1136 (41.2)		
Overweight (24.99 > BMI < 30.00)	880 (32.2)	953 (34.6)		
Obese (BMI > 29.99)	436 (15.9)	593 (21.5)		
Employment			178.29 (6)	< 0.001
Full-time	1145 (38.0)	1123 (37.0)		
Part-time	463 (15.4)	541 (17.8)		
Student	270 (9.0)	277 (9.1)		
Unemployed/Home duties	620 (20.6)	325 (10.7)		
Retired	460 (15.3)	607 (20.0)		
Other	53 (1.8)	161 (5.3)		

*Income and BMI data provided by 82.4% and 90.9% of participants respectively; *BMI* body mass index.

### Objective binge eating

#### Prevalence

**Figure 1:**
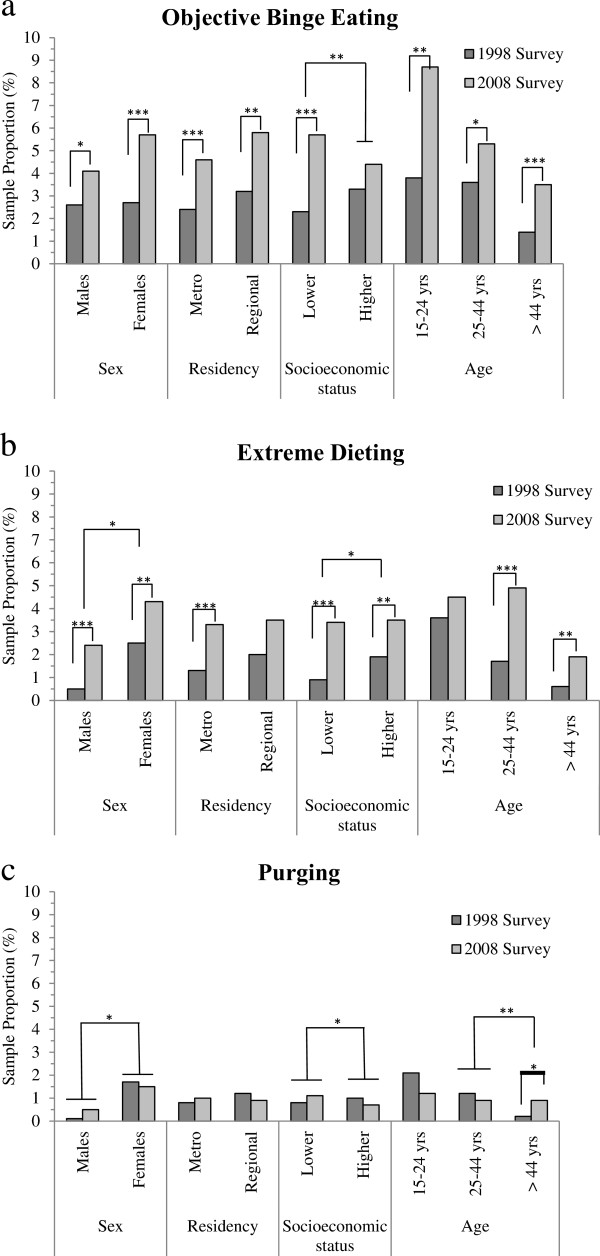
**The prevalence of eating disorder behaviors within demographic sectors in the 1998 and 2008 surveys.** **a**. Prevalence of objective binge eating within demographic sectors in the 1998 and 2008 surveys. **b**. Prevalence of extreme dieting within demographic sectors in the 1998 and 2008 surveys. **c** Prevalence of purging within demographic sectors in the 1998 and 2008 surveys. Significant differences within demographic sectors between survey years (e.g. males in 1998 vs. males in 2008) indicate the results of χ^2^ tests. Significant differences between demographic sectors over time (e.g. males vs. females) indicate the results of the Altman and Bland method to compare odds ratios derived from ordinal logistic regressions. *p < 0.05, **p < 0.01, ***p < 0.001.

The proportion of participants reporting eating disorder behaviors within demographic sectors in each survey is illustrated in Figure 1. As can be seen in Table 2, the prevalence of objective binge eating was higher in 2008 compared to 1998 in both men and women, within all age groups, and in both metropolitan and regional areas. Objective binge eating however was observed to be significantly higher in 2008 compared to 1998 in the sector of participants who earned below the median household income but not those who earned above it. Using the Altman and Bland method, the odds ratios calculated across 1998 and 2008 data for objective binge eating were found not to differ significantly between men and women, between any of the age groups, or between metropolitan and regional areas, indicating a similar rate of increase across sex, age, and residency (all *p* > 0.05). However the odds ratio was higher for participants below the median household income level (*z* = 2.69, *p* = 0.01), indicating that objective binge eating is increasing at a significantly faster rate in the lower socioeconomic sector.

**Table 2:** **Comparison of the prevalence of eating disorder behaviors in 1998 and 2008: results of Chi-Square Tests and multivariate logistic regressions**

	1998 (*n* = 3010)	2008 (*n* = 3034)				
	*n* (%)	*n* (%)	χ ^2^	*P*	*OR* (95% *CI* )†	*P*
Objective binge eating						
Males	38 (2.6)	61 (4.1)	5.29	0.02	1.91 (1.17 – 3.10)	0.01
Females	42 (2.7)	88 (5.7)	16.71	< 0.001	2.53 (1.61 – 3.96)	< 0.001
Metropolitan	50 (2.4)	103 (4.6)	15.05	< 0.001	1.74 (1.15 – 2.64)	0.01
Regional	30 (3.2)	46 (5.8)	6.82	0.01	1.95 (1.04 – 3.67)	0.04
< median income	26 (2.3)	72 (5.7)	17.78	< 0.001	3.29 (1.88 – 5.77)^c^	< 0.001
> median income	47 (3.3)	51 (4.4)	2.28	0.13	1.26 (0.83 – 1.92)	0.28
15-24 years	20 (3.8)	43 (8.7)	10.31	0.00	2.81 (1.39 – 5.69)	0.00
25-44 years	41 (3.6)	53 (5.3)	3.95	0.05	1.39 (0.86 – 2.23)	0.18
≥ 45 years	19 (1.4)	54 (3.5)	12.53	< 0.001	1.90 (1.04 – 3.46)	0.04
Extreme dieting						
Males	8 (0.5)	35 (2.4)	16.96	< 0.001	4.87 (2.14 – 11.09)^a^	< 0.001
Females	38 (2.5)	66 (4.3)	7.69	0.01	1.69 (1.09 – 2.63)	0.02
Metropolitan	27 (1.3)	73 (3.3)	18.27	< 0.001	2.76 (1.64 – 4.65)^b^	< 0.001
Regional	19 (2.0)	28 (3.5)	3.69	0.06	1.25 (0.57 – 2.74))	0.57
< median income	10 (0.9)	42 (3.4)	16.89	< 0.001	6.31 (2.64 – 15.08)^c^	< 0.001
> median income	27 (1.9)	40 (3.5)	6.36	0.01	1.85 (1.13 – 3.05)	0.02
15-24 years	19 (3.6)	22 (4.5)	0.43	0.51	1.67 (0.70 – 3.96)	0.25
25-44 years	20 (1.7)	49 (4.9)	17.53	< 0.001	2.52 (1.46 – 4.37)	0.00
≥ 45 years	8 (0.6)	30 (1.9)	9.99	0.00	4.43 (1.68 – 11.70)	0.00
Purging						
Males	2 (0.1)	7 (0.5)	2.75	0.10	6.15 (1.22 – 31.10)^a^	0.03
Females	26 (1.7)	23 (1.5)	0.21	0.65	0.93 (0.48 – 1.80)	0.83
Metropolitan	17 (0.8)	22 (1.0)	0.32	0.57	1.59 (0.75 – 3.37)	0.23
Regional	11 (1.2)	7 (0.9)	0.35	0.55	0.64 (0.13 – 3.18)	0.58
< median income	9 (0.8)	14 (1.1)	0.62	0.43	4.28 (1.20 – 15.26)^c^	0.03
> median income	15 (1.0)	8 (0.7)	0.91	0.34	0.80 (0.34 – 1.89)	0.61
15-24 years	11 (2.1)	6 (1.2)	1.22	0.27	1.88 (0.42 – 8.33)	0.41
25-44 years	14 (1.2)	9 (0.9)	0.49	0.49	0.55 (0.21 – 1.49)	0.24
≥ 45 years	3 (0.2)	14 (0.9)	5.70	0.02	9.48 (1.21 – 74.51)^d^	0.03

*OR* odds ratio; † multivariate logistic regressions included age, education, income, body mass index, employment, and country of birth as covariates; ^a^the OR was significantly greater for males than for females; ^b^the OR was marginally greater for metropolitan versus regional participants; ^c^the OR was significantly greater for participants below compared to above the median income level; ^d^the OR was significantly greater for participants aged over 44 years compared to those aged 25 – 44 years.

#### Quality of life

See Table 3 for the means and standard deviations of the SF-36 scores according to survey year, reported ED behaviors, and demographic variables. A significant main effect of survey year was found on the PCS scores of participants who reported objective binge eating (*F* (1, 182) = 9.19, *p* = 0.00), with scores being higher in 2008 compared to 1998. A significant sex by survey year interaction (*F* (1, 187) = 5.43, *p* = 0.02) was found on the MCS scores of participants who reported objective binge eating, with scores decreasing between 1998 and 2008 for males but remaining stable for females. No main effects of sex or interactions between sex and survey year were significant on the PCS scores, indicating an overall similar level of physical health-related quality of life impairment associated with objective binge eating between men and women. A main effect of residency (*F* (1, 182) = 3.80, *p* = 0.05) was found on scores of the PCS in participants who reported objective binge eating, with regional participants scoring lower than metropolitan participants. No main effects of residency or interactions between residency and survey year were found on the MCS, indicating an overall similar level of mental health-related quality of life impairment associated with objective binge eating in metropolitan and regional areas. No main effects of annual household income or age, or interactions between these demographic variables and survey year were found on the MCS or PCS scores of participants who reported objective binge eating, indicating an overall similar level of mental and physical health-related quality of life impairment associated with objective binge eating across income and age groups.

**Table 3:** **Health-related quality of life associated with eating disorder behaviors according to year, sex, age, residency, and household income**

	Sex		Age in years			Residency		Household income	
	Male	Female	15 – 24	25 - 44	≥ 45	Metro	Regional	< Median	> Median
**1998 – PCS scores,** ***M*** **(** ***SD*** **)**									
Objective binge eating	49.39 (11.89)	45.05 (14.08)	51.33 (10.53)	46.54 (12.98)	37.42 (15.57)	45.67 (13.47)	40.95 (17.10)	40.88 (14.63)	49.59 (11.24)
Extreme dieting	48.64 (9.12)	47.23 (9.65)	50.91 (8.78)	49.20 (9.24)	40.64 (13.19)	48.99 (9.96)	44.08 (12.27)	51.53 (11.42)	47.05 (7.01)
Purging	42.39 (12.84)	48.61 (8.14)	47.53 (10.42)	51.38 (8.40)	38.93 (13.12)	49.91 (9.95)	44.15 (9.80)	46.07 (11.09)	48.32 (7.24)
**2008 – PCS scores,** ***M*** **(** ***SD*** **)**									
Objective binge eating	49.39 (11.89)	45.05 (14.08)	51.53 (6.41)	48.18 (7.74)	44.00 (11.13)	48.42 (8.70)	43.52 (11.00)	46.02 (9.97)	49.00 (8.52)
Extreme dieting	48.33 (10.21)	48.40 (9.59)	49.93 (7.01)	51.60 (8.24)	42.37 (11.82)	48.79 (9.70)	47.78 (11.35)	46.71 (11.10)	50.05 (8.30)
Purging	48.22 (10.50)	46.71 (7.87)	50.46 (8.80)	47.08 (10.03)	46.54 (9.54)	46.91 (8.66)	48.25 (12.40)	47.78 (8.75)	51.34 (4.82)
**1998 – MCS scores,** ***M*** **(** ***SD*** **)**									
Objective binge eating	46.35 (13.67)	38.87 (14.42)	36.21 (11.37)	40.27 (12.97)	39.18 (16.83)	38.55 (13.36)	42.51 (15.98)	39.96 (12.18)	44.50 (15.36)
Extreme dieting	51.84 (5.34)	45.00 (12.18)	47.71 (9.53)	43.87 (14.61)	42.23 (10.96)	44.63 (13.01)	44.62 (11.05)	42.40 (12.85)	46.78 (11.93)
Purging	48.52 (12.24)	48.55 (11.92)	40.36 (13.33)	46.65 (12.49)	52.34 (16.07)	44.24 (12.93)	47.86 (15.12)	51.26 (9.87)	46.63 (12.30)
**2008 – MCS scores,** ***M*** **(** ***SD*** **)**									
Objective binge eating	41.21 (14.71)	42.66 (13.16)	40.91 (12.34)	41.20 (14.30)	43.34 (15.94)	42.14 (14.85)	41.90 (14.01)	38.49 (14.40)	44.25 (12.87)
Extreme dieting	43.08 (15.04)	41.09 (13.73)	41.84 (12.26)	41.85 (13.05)	38.93 (17.58)	40.89 (14.03)	41.31 (15.64)	37.51 (16.62)	45.53 (11.28)
Purging	48.74 (9.63)	42.19 (11.33)	43.33 (12.73)	43.31 (13.11)	45.92 (10.65)	44.72 (12.19)	43.82 (10.03)	43.68 (9.89)	46.44 (7.96)

*PCS* physical health composite scale, Medical Outcomes Study Short Form (SF-36); *MCS* mental health composite scale, SF-36.

### Extreme dieting

#### Prevalence

As can be seen in Table 2, from 1998 to 2008 the prevalence of extreme dieting increased significantly in both men and women and in both the below and above median household income sectors. Extreme dieting only increased in the 25–44 and ≥ 45 year age groups however, and not in the 15–24 year age group. Extreme dieting also only increased in metropolitan but not regional areas. The 1998–2008 odds ratio was significantly greater in men versus women (*z* = −2.22, *p* = 0.03) and in the below versus above median income group (*z* = 2.40, *p* = 0.02), indicating a greater rate of increase of extreme dieting in males and the lower socioeconomic sector. On the other hand, the 1998–2008 odds ratios did not differ significantly between any of the age groups (all *p* > 0.05), indicating a similar rate of increase in these behaviors across age. The difference between odds ratios based on residency approached but did not reach significance (*z* = 1.65, *p* = 0.10), with a non-significant greater rate of increase of extreme dieting found in the metropolitan areas.

#### Quality of life

No main effects of survey year were found for scores on the PCS or MCS for participants who reported extreme dieting. A significant interaction between annual household income and survey year (*F* (1, 108) = 3.91, *p* = 0.05) was found on scores of the PCS in participants who reported extreme dieting. While scores decreased between 1998 and 2008 within the group who earned below the median household income, scores increased within the group who earned above the median household income. No main effects of annual household income or interactions between annual household income and survey year were found on the MCS scores for participants who reported extreme dieting. No main effects of sex, age, or residency, or interactions between these demographic variables and survey year were significant on the PCS or MCS scores of participants who reported extreme dieting, indicating an overall similar level of physical and mental health-related quality of life impairment associated with extreme dieting across sex, age groups, and regional vs. metropolitan residency.

### Purging

#### Prevalence

As can be seen in Table 2, from 1998 to 2008, purging significantly increased in the ≥ 45 year age group but not the younger age groups. There was no significant difference in the prevalence of purging in 1998 vs. 2008 however in either men or women, participants who earned below or above the median household income, or participants living within regional or metropolitan areas. In comparing the 1998–2008 odds ratios between demographic sectors however, the odds ratios were significantly greater for the ≥ 45 year group vs. the 25–44 year age group (*z* = 2.45, *p* = 0.01), for men vs. women (*z* = 2.12, *p* = 0.03), and for participants who earned below vs. above the median income (*z* = 2.14, *p* = 0.03). This suggests that purging is increasing at a faster rate in older age groups, males, and the lower socioeconomic sector. No significant difference was observed between the 1998–2008 odds ratios in the regional vs. metropolitan areas, indicating that purging is increasing at a similar rate regardless of residency.

#### Quality of life

No main effects of survey year were found for scores on the PCS or MCS for participants who reported purging. No main effects of any of the demographic variables assessed (i.e. sex, age, residency, and household income) or interactions between these variables and survey year were significant on the PCS or MCS scores of participants who reported purging, indicating an overall similar level of physical and mental health-related quality of life impairment associated with purging across sex, age, residency, and socioeconomic status.

## Discussion

This study provided the first known investigation into demographic differences in the prevalence and associated impairment of ED behaviors over time. This was achieved by comparing the proportion of participants who reported behaviors in surveys conducted on samples of the South Australia population in 1998 and 2008. Overall the findings from this study indicated that ED behaviors increased most rapidly from 1998 to 2008 in those demographic sectors that were previously characterized as being less eating disordered. For instance, binge eating, extreme dieting, and purging (self-induced vomiting, laxative and diuretic misuse) all increased at a faster rate in participants who lived in households that earned below the median annual income. The prevalence of extreme dieting and purging also increased at a faster rate in men compared to women. Furthermore, purging behaviors increased at the fastest rate in participants from the oldest age group, aged over 44 years. These findings offer clear counter-evidence to the historic stereotype that EDs are suffered by young upper-class females.

For some time it has been acknowledged in the literature that of all the ED symptoms, objective binge eating may be as common in men as it is in women [5, 8, 19]. Our findings demonstrate that in addition men may also be ‘catching up’ to women in the prevalence of severe weight and shape control symptoms, such as the dietary practice of fasting for long hours and the purging practices of self-induced vomiting and laxative abuse. The increased prevalence of purging that was also found in the older age group of the current study appears to represent a new phenomenon, as this behavior was virtually non-existent in this age group in 1998. The 2008 prevalence however approximated that seen in the younger age groups. This finding is supported by a recent cross-sectional study of a 2011 survey that reported 5-year self-reported prevalence rates for purging of 5.9 – 8.1% in women aged 50 – 84 years [10].

Overall, ED behaviors were associated with a similar level of health-related quality of life impairment across demographic variables, suggesting that the impact of these behaviors is detrimental regardless of sex, age, socioeconomic status, and residency. Some effects related to the impact of binge eating and dieting did emerge however. Objective binge eating was associated with greater physical health-related quality of life impairment in participants from regional (vs. urban) areas and a worsening of mental health-related quality of life impairment over time in men compared to women. Extreme dieting was associated with a worsening of physical health-related quality of life impairment in participants below the median annual household income. These findings may suggest that while the prevalence of disordered eating is evening out across demographics, the rate of increase and its impact on perceived functioning remains highest for marginalized groups with less access to specialized care, such as those who are poorer and live outside of the major cities. Further, the portrayal of EDs in the media and health promotion efforts as being problems that predominantly affect women may have inadvertently contributed to increased stigma associated with disordered eating in men. These issues, the inadequate access to resources and narrow demographic target of existing resources (i.e. young females), could partly explain less treatment seeking and under-detection of EDs in males [3, 4] and other under-represented sectors of the community*.* Ultimately this may have contributed to the increased prevalence and associated health-related quality of life impairment found in this study amongst males and older, regional, and poorer people.

Study limitations were that other core ED behaviors were not assessed across the survey years, such as compulsive exercise and subjective binge eating. This would be of interest in future studies, as some of these behaviors may be particularly relevant to demographic sectors (e.g. exercise as a compensatory behavior for males with EDs [33]). Ethnicity data was not available in the present study, and understanding how the prevalence and impact of ED symptoms is changing within ethnic groups will also be an important endeavor for future research. This study was conducted using two sequential cross-sectional surveys, which although using a very stringent selection process, meant that only differences between the samples rather than within-sample changes could be assessed. Further, although efforts were made to control for sample differences in the demographics that were measured, other possible influential factors were not measured (e.g. acculturation and generational status) and so could not be controlled in analyses. A replication of this study using a longitudinal sample would provide an account of changes within one representative sample and greater confidence of actual temporal trends in the population. Finally, a few of the analyses conducted were based on relatively small *n*’s (especially those concerning purging behavior), which reduces confidence in the reliability of these particular results.

The finding that ED behaviors are increasing most rapidly in those demographic sectors where disordered eating was previously least prevalent has implications for how public campaigns on EDs are addressed. If the present findings are an indication for the current and emerging demographic profile of EDs, it is imperative that more gender- and age-neutral, or specialized, health promotion strategies are developed. The ED field also must actively attempt to decrease demographic bias in the selection of research participants [34], by recruiting male and female participants from a representative range of socioeconomic, age, geographic, and ethnic backgrounds. Last but not least, it is important that access to services is improved for disadvantaged groups, such as those from low income and less urbanized communities.

## Conclusion

This study found that disordered eating increased over the decade 1998 to 2008 across all demographic sectors, but at a faster rate in male, lower socioeconomic, and older participants. Further, the burden of disordered eating, indicated by impairment in health-related quality of life, was greatest in underserved groups, including participants from a lower socioeconomic background and who live in regional areas. This provides further evidence to suggest that disordered eating and EDs may exist across the demographic spectrum and are not specific to young females. Consequently, treatment and prevention programs should be developed to target the wider community and ED research should aim to recruit demographically representative samples.

## Electronic supplementary material

Additional file 1: **Interview questions to elicit eating disorder behaviours in the 1998 and 2008 Health Omnibus Surveys.** (DOC 44 KB)

## Abbreviations

ED: Eating disorder
SF-36: Medical outcomes study short form
PCS: Physical health composite scale
MCS: Mental health composite scale
MANOVA: Multivariate analyses of variance.
