# Supplementary material for: The changing demographic profile of eating disorder behaviors in the community
Source: BMC Public Health. 2014 Sep 11;14:943. doi: 10.1186/1471-2458-14-943 (PMC4246495; doi:10.1186/1471-2458-14-943)
Supplement: Supplementary file 1 — Additional file 1: Interview questions to elicit eating disorder behaviours in the 1998 and 2008 Health Omnibus Surveys. (DOC 44 KB) [file 12889_2013_7297_MOESM1_ESM.doc]

| NOTE: The structured questions below regarding the experience of eating disorder behaviors were included as part of the Health Omnibus Surveys in 1998 and 2008. Interviewers were instructed to read the questions verbatim (words in bold text) and record the participant’s response. | |
| --- | --- |
| **BEHAVIOR** | QUESTION |
| Objective binge eating | **I would now like to ask you about episodes of overeating. By overeating, or binge eating, I mean eating an unusually large amount of food in one go and at the time feeling that your eating was out of control.** Interviewer note respondent could not prevent themselves from overeating, or could not stop eating once they had started.  Over the past three months how often have you overeaten? Would you say…   1. **Not at all** 2. **Less than weekly** 3. **Once a week** 4. **Two or more times a week** 5. Don’ t know 6. Refused |
|  | The next questions are about various weight-control methods some people use. |
| Purging | Over the past three months have you regularly used, that is at least once a week, any of the following: laxatives, diuretics (water tablets), made yourself sick, in order to control your shape or weight?   1. Yes 2. No 3. Refused |
| Extreme dieting | Over the past three months have you regularly done any of the following: gone on a very strict diet, or eaten hardly anything at all for a time, in order to control your shape or weight? Interview note: at least once weekly, or recurrently during the three months   1. Yes 2. No 3. Refused |
